# The Interaction Between the *asb5a* and *asb5b* Subtypes Jointly Regulates the L-R Asymmetrical Development of the Heart in *Zebrafish*

**DOI:** 10.3390/ijms26062765

**Published:** 2025-03-19

**Authors:** Wanbang Zhou, Wanwan Cai, Yongqing Li, Luoqing Gao, Xin Liu, Siyuan Liu, Junrong Lei, Jisheng Zhang, Yuequn Wang, Zhigang Jiang, Xiushan Wu, Xiongwei Fan, Fang Li, Lan Zheng, Wuzhou Yuan

**Affiliations:** 1 The Laboratory of Heart Development Research, College of Life Science, Hunan Normal University, Changsha 410081, China; zwb@hunnu.edu.cn (W.Z.); 202403022@hunnu.edu.cn (W.C.); liyongqing2002@hunnu.edu.cn (Y.L.); 202220142836@hunnu.edu.cn (L.G.); 202220142838@hunnu.edu.cn (X.L.); 202220142830@hunnu.edu.cn (S.L.); yuequnwang@hunnu.edu.cn (Y.W.); 201201140149@hunnu.edu.cn (Z.J.); xiushanwu2003@aliyun.com (X.W.); 16119@hunnu.edu.cn (X.F.); lifang@hunnu.edu.cn (F.L.); 2 Laboratory of Physical Fitness and Exercise Rehabilitation of Hunan Province, College of Physical Education, Hunan Normal University, Changsha 410012, China; 11238@hunnu.edu.cn (J.L.); 14909@hunnu.edu.cn (J.Z.)

**Keywords:** *asb5a*, *asb5b*, left–right asymmetric, cardiac looping, nodal signaling pathway

## Abstract

The *asb5* gene, a member of the Asb protein subfamily characterized by six ankyrin repeat domains, is highly conserved and comprises two subtypes, *asb5a* and *asb5b*, in *zebrafish*. Our previous research has demonstrated that a deficiency of the *asb5* gene significantly impairs early cardiac contractile function, highlighting its close relationship with heart development. *Zebrafish asb5* expression was disrupted by both morpholino (MO) antisense oligomer-mediated knockdown and a CRISPR-Cas9 system. A high-throughput RNA-Seq analysis was used to analyze the possible molecular regulatory mechanism of *asb5* gene deletion leading to left–right (L-R) asymmetry defects in the heart. Whole-mount in situ hybridization (WISH) was conducted to evaluate gene expression patterns of Nodal signaling components and the positions of heart organs. Heart looping was defective in *zebrafish asb5* morphants. Rescue experiments in the *asb5*-deficiency group (inactivating both *asb5a* and *asb5b*) demonstrated that the injection of either *asb5a*-mRNA or *asb5b*-mRNA alone was insufficient to rectify the abnormal L-R asymmetry of the heart. In contrast, the simultaneous injection of both *asb5a*-mRNA and *asb5b*-mRNA successfully rescued the morphological phenotype. A high-throughput RNA-Seq analysis of embryos at 48 h post fertilization (hpf) revealed that numerous genes associated with L-R asymmetry exhibited expression imbalances in the *asb5*-deficiency group. WISH further confirmed that the expression of genes such as *fli1a*, *acta1b*, *hand2*, *has2*, *prrx1a*, *notch1b*, and *foxa3* were upregulated, while the expression of *mei2a* and *tal1* was downregulated. These results indicated that loss of the *asb5* gene in *zebrafish* led to the disordered development of L-R asymmetry in the heart, resulting in an imbalance in the expression of genes associated with the regulation of L-R asymmetry. Subsequently, we examined the expression patterns of classical Nodal signaling pathway-related genes using WISH. The results showed that the midline barrier factor gene *lefty1* was downregulated at early stages in the *asb5*-deficiency group, and the expression of *spaw* and *lefty2*, which are specific to the left lateral plate mesoderm (LPM), was disrupted. This study reveals that the two subtypes of the *asb5* gene in *zebrafish*, *asb5a* and *asb5b*, interact and jointly regulate the establishment of early cardiac L-R asymmetry through the Nodal-*spaw*-*lefty* signaling pathway.

## 1. Introduction

Nearly all thoracic and abdominal organs of vertebrates exhibit L-R asymmetry in their anatomy, placement, and, in some cases, physiology. This pronounced L-R asymmetry of visceral organs is recognized as being dependent on left- and right-side-specific gene expression cascades during early embryogenesis [1]. The proper establishment of the L-R axis is crucial for organogenesis in vertebrates [2]. Although the asymmetric patterns of gene expression vary among species, a conserved feature across all examined animal species is the production of the secreted TGFβ-like factor, Nodal (or a Nodal homolog), in the left lateral plate mesoderm [3]. Nodal, a ligand within the TGFβ protein family, serves as a primary regulator of L-R axis establishment. Following its role in mesoderm patterning, *nodal* is expressed in the embryonic node and the left lateral plate mesoderm in species such as mouse, *zebrafish*, chick and *Xenopus* [4,5,6]. In *zebrafish*, the first identified Nodal homolog gene is *southpaw* (*spaw*), which encodes a novel member of the *nodal*-related class of proteins, a subfamily within the transforming growth factor β superfamily of secreted factors [7]. *Spaw* is expressed in the left LPM to activate the Nodal pathway within the left side of the heart [8]. The downstream targets of *spaw* include *pitx2*, *lefty1* and *lefty2* [9]. *Lefty1* is expressed at the midline in *zebrafish* and functions as a `molecular midline barrier’, preventing the propagation of Nodal from the left to the right LPM [10]. The absence of *lefty1* results in bilateral expression of *nodal*, *lefty2*, and *pitx2* in mice [11]. At early somite stages, *nodal* and *lefty2* are co-expressed in the left lateral plate mesoderm [12]. The regulation of Nodal signaling also involves negative feedback, whereby Nodal ligands induce the synthesis of their own antagonists, the Lefty1 and Lefty2 proteins [7,12]. In vertebrate embryos, the typical lateralization of the heart loop is known as rightward or dextro-looping (D-loop). Conversely, the abnormal lateralization process that results in a mirror-image configuration is termed the leftward or levo-looping (L-loop). Additionally, the linearization of the heart is described as no-looping (N-loop). Numerous studies have demonstrated that the laterality of the heart loop, as well as that of other developing organs, is regulated by the molecular L-R signaling cascades previously mentioned [13,14,15].

The Asb family members exhibit a relatively divergent N-terminal domain, followed by a variable number of ankyrin repeats and a C-terminal SOCS box [16]. Of particular interest is the subfamily of six-ankyrin repeat domain-containing Asb proteins, which includes Asb5, Asb9, Asb11, and Asb13. These proteins exhibit remarkable evolutionary conservation and play a role in mediating compartment size expansion, such as regulating the sizes of the brain and muscle compartments [17]. Currently, the biological functions of Asb5 remain poorly understood. However, it has been reported that Asb5 is a novel protein implicated in the initiation of arteriogenesis [18]. In *zebrafish*, *asb5* is categorized into two subtypes: *asb5a* and *asb5b*. The *asb5a* is located on chromosome 14 and comprises 6 exons and 5 introns, with a full-length cDNA of 915 bp that encodes 232 amino acids. The Asb5a protein features three evolutionarily conserved ANK functional domains and one SOCS-box domain. In contrast, *asb5b* is situated on chromosome 1 and comprises 7 exons and 6 introns, with a full-length cDNA of 1545 bp that encodes 328 amino acids. The Asb5b protein contains six evolutionarily conserved ANK functional domains and one SOCS-box domain.

Our previous work revealed that the early expression patterns of the two subtypes, *asb5a* and *asb5b*, are similar. Both are broadly expressed starting from the high cap stage of embryonic development [19], suggesting that they may play regulatory roles in early embryonic development. After completely knocking out the *asb5a* gene in *zebrafish* using CRISPR/Cas9 technology [20,21], the homozygous embryos did not show any abnormalities. Using morpholino interference technology [22,23] to further knock down the expression of *asb5b* in *asb5a*^-*/*-^ *zebrafish* resulted in abnormal cardiac contraction function.

In this study, we found that at the 48 hpf embryo stage, inactivating both *asb5a* and *asb5b* (the *asb5*-deficiency group) resulted in an abnormal phenotype characterized by cardiac L-R asymmetry compared to other groups. The heart exhibited variations in looping, which were categorized as normal right looping, non-looping, and reversed left looping. We confirmed that the two subtypes of *asb5*, *asb5a*, and *asb5b*, interact genetically and jointly regulate the establishment of early cardiac L-R asymmetry patterns via the Nodal-*spaw*-*lefty* signaling pathway.

## 2. Results

### 2.1. Loss of asb5 Disrupts Heart L-R Asymmetry Development in Zebrafish Embryos

**Figure 1:**
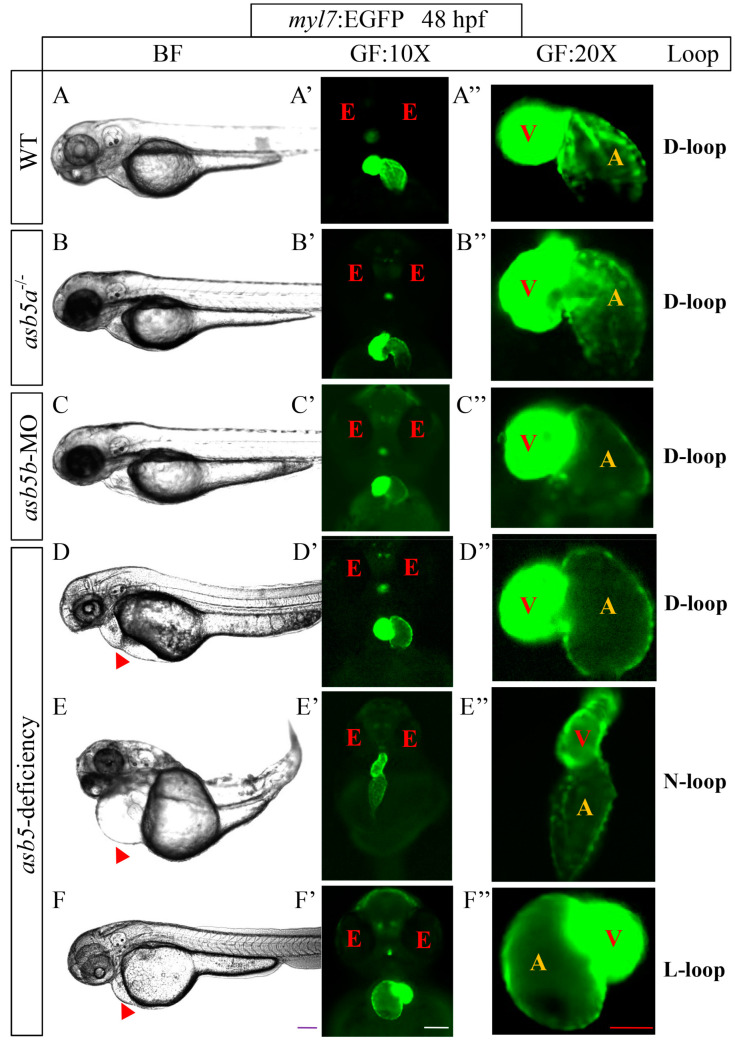
The impact of *asb5a/asb5b* deficiency on L-R asymmetry in cardiac development. (**A**–**F**) Representative images of the pericardial cavity in various groups at 48 hpf during embryonic development; scale bar = 200 µm (side view). (**A′**–**F′**) Representative images of heart positioning in different *zebrafish* groups at 48 hpf under the *myl7*:EGFP background; scale bar = 100 µm (belly up view); (**A″**–**F″**) Magnified images of the atria and ventricles in various *zebrafish* groups at 48 hpf, also under the *myl7*:EGFP background, scale bar = 50 µm (belly up view). The red triangular arrows indicate areas of enlargement in the cardiac pericardial cavity. BF: bright field; GF: green fluorescence; 10X: field of view under a 10× microscope; 20X: field of view under a 20× microscope; red E: eyes; red V: ventricle; yellow A: atrium; D-loop: dextral looping; N-loop: no looping; L-loop: levo-looping.

**Figure 2:**
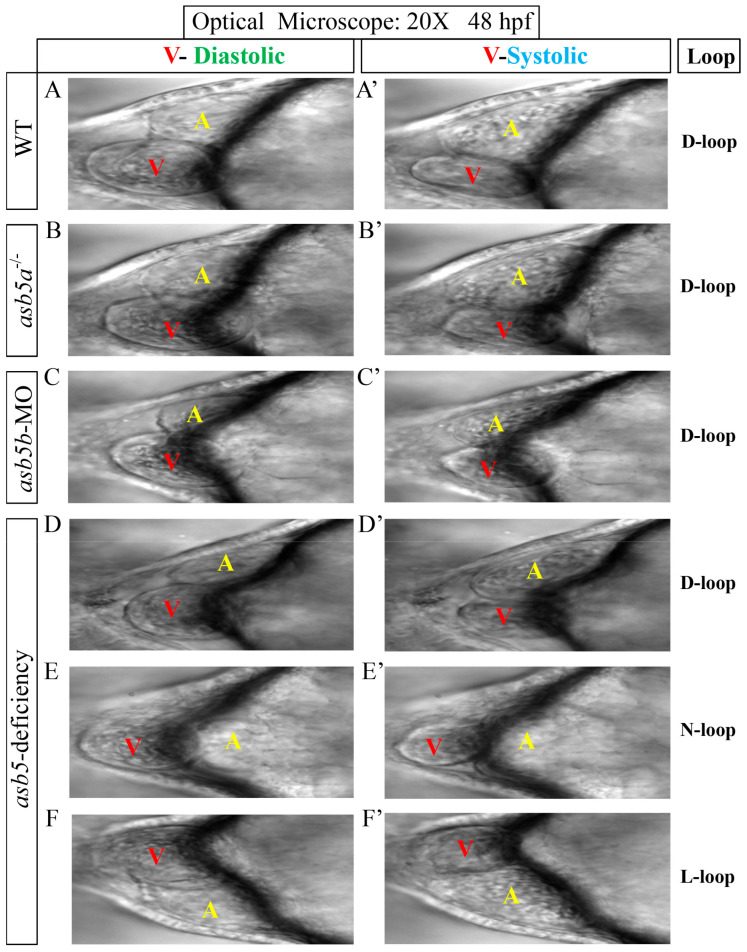
The morphological changes following the inactivation of *asb5a*/*asb5b*. The 10 s M-mode screenshot clearly displays the diastolic and systolic shapes of the ventricle and atrium. (**A**–**F**) Schematic diagrams of cardiac looping during diastole of the ventricle in various groups of *zebrafish* at 48 hpf. (**A′**–**F′**) Schematic diagrams of cardiac looping during ventricular systole in these same groups of *zebrafish* at 48 hpf. 20X: field of view under a 20× microscope; red V: ventricle; yellow A: atrium; D-loop: dextral looping; N-loop: no looping; L-loop: levo-looping.

As mentioned in our previous article [19], we successfully generated *asb5a*^-*/*-^ knockout homozygous line, *asb5b*-MO knockdown line, and *asb5*-deficiency line (simultaneous knockdown of *asb5b* in *asb5a*^-/-^ homozygous embryos line) in *zebrafish* using CRISPR/Cas9 knockout technology and morpholino interference technology (Figures S4 and S5). By combining with the *myl7*:EGFP transgenic *zebrafish* line, which specifically expresses green fluorescent protein in cardiac tissue [24], we observed that the embryos in the *asb5*-deficiency group at the 48 hpf stage exhibited pericardial edema (indicated by the red triangular arrow in Figure 1) and abnormal cardiac looping. Three distinct patterns of cardiac looping were identified: D-loop, N-loop, and L-loop (Figure 1). Subsequently, we employed a high-speed EM-CCD camera to capture a 10 s video at 130 frames per second and analyzed the recorded footage using the Semi-automatic Heartbeat Analysis software program (Version 3.4.0.0) [25]. By extracting individual frames from the video (each measuring 1300 × 600 pixels), we further confirmed that the *asb5*-deficiency group exhibited abnormal looping phenotypes (Figure 2). These findings suggest that the concurrent loss of *asb5a* and *asb5b* subtypes in *zebrafish* disrupts the normal pattern of L-R asymmetry during early heart development.

### 2.2. WISH Results Indicating Loss of asb5 Leads to Abnormal Cardiac Looping

**Figure 3:**
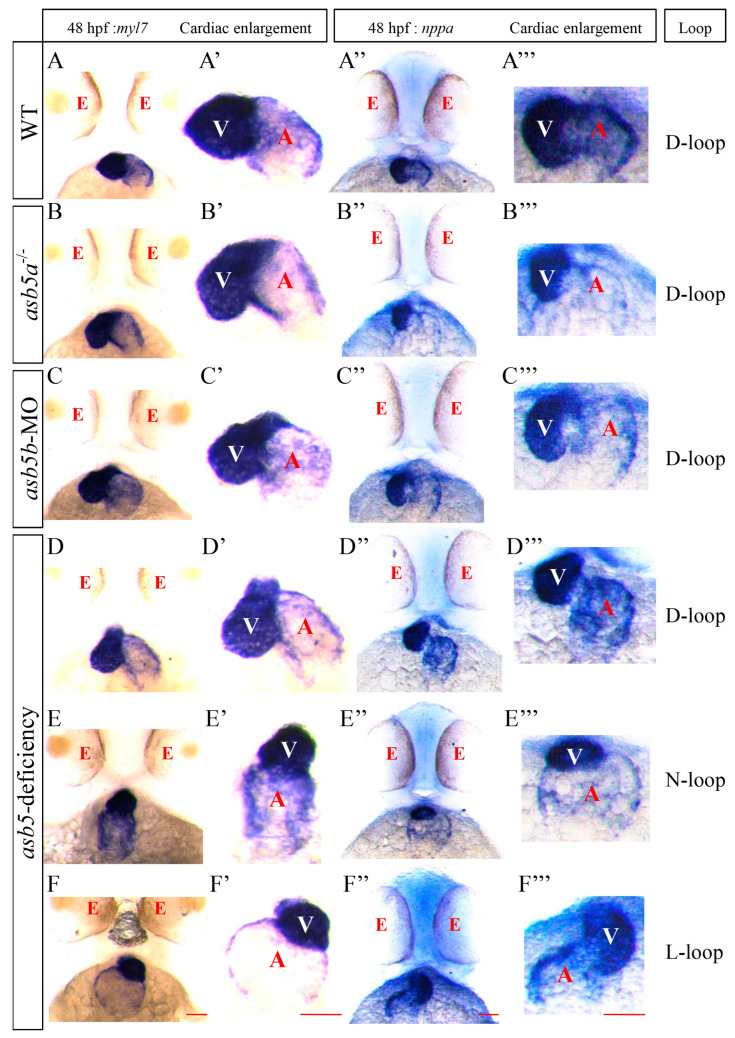
WISH analysis of heart looping at 48 hpf in the *asb5*-deficiency group, utilizing *myl7* and *nppa* probes. (**A**–**F**) Schematic diagrams of heart positions marked with *myl7* probes in different groups at 48 hpf. (**A′**–**F′**) Enlarged views of these heart positions as indicated by *myl7* probes. (**A″**–**F″**) Schematic diagrams of heart positions marked with *nppa* probes in various groups at 48 hpf. (**A′′′**–**F′′′**) present enlarged views of these positions as indicated by *nppa* probes. Scale bar = 100 µm (belly up view). red E: eyes; white V: ventricle; red A: atrium; D-loop: dextral looping; N-loop: no looping; L-loop: levo-looping.

To ascertain that the L-R asymmetry defects observed in the *asb5*-deficient group are not merely coincidental or induced by environmental factors, we employed WISH to analyze the expression of heart-specific genes *myl7* (*myosin*, *light chain 7*) [26] and *nppa* (*natriuretic peptide A*) [27] in *zebrafish* embryos at 48 hpf. Our findings revealed that, at 48 hpf, the *asb5*-deficiency group exhibited three distinct patterns of cardiac looping: normal D-loop, non-looping N-loop, and reverse L-loop (Figure 3). This finding suggests that the disruption of cardiac asymmetry patterns observed in *asb5*-deficient *zebrafish* at 48 hpf is not a chance occurrence, further highlighting the crucial role of the *asb5* gene in establishing early cardiac asymmetry patterns.

### 2.3. The Atria and Ventricles in the asb5-Deficiency Group Are Normally Differentiated

**Figure 4:**
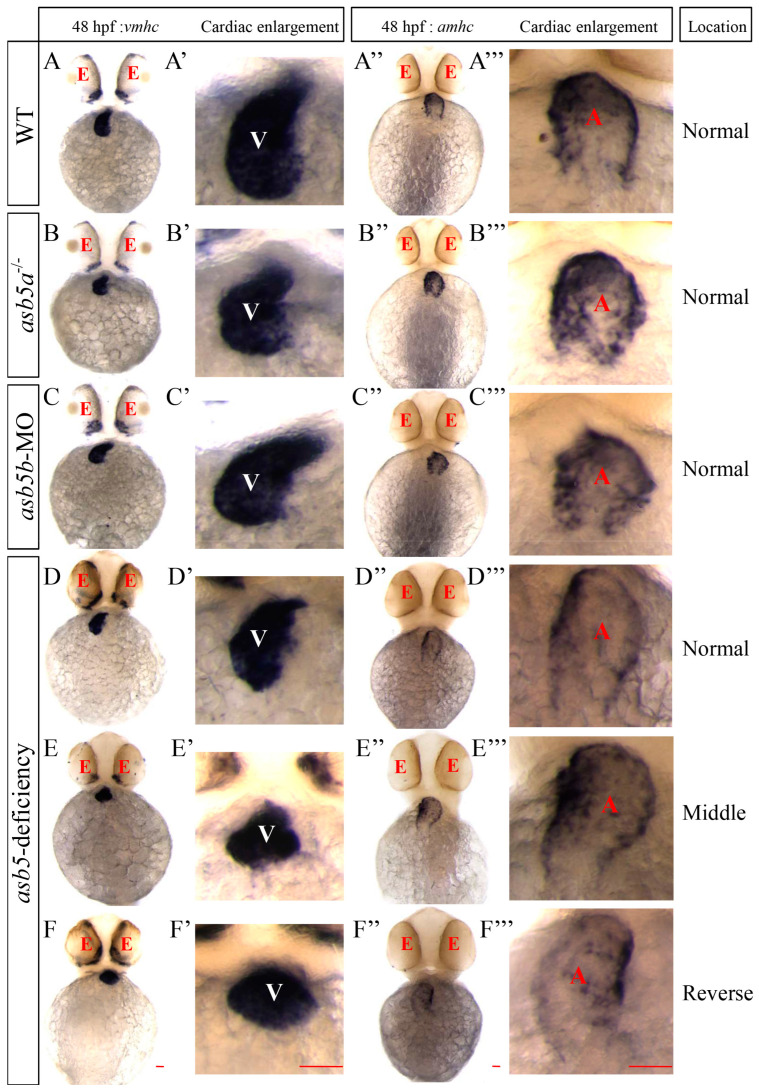
WISH results using *vmhc* and *amhc* probes at 48 hpf in *zebrafish* embryos. (**A**–**F**) Schematic representations of ventricular positions labeled by the *vmhc* probe across different groups at 48 hpf. (**A′**–**F′**) Enlarged images of ventricular tissues labeled by the *vmhc* probe in these groups. (**A″**–**F″**) Schematic representation of the atrial positions labeled by the *amhc* probe across various groups at 48 hpf. (**A′′′**–**F′′′**) Enlarged images of atrial tissues labeled by the *amhc* probe in these groups. red E: eyes; white V: ventricle; red A: atrium; Normal: Normal position; Middle: Middle position; Reverse: Reverse position; Scale bar = 100 µm (belly up view).

To determine whether the observed cardiac asymmetry defect in the *asb5*-deficiency group was due to defects in the differentiation of ventricular or atrial tissue, WISH was performed on 48 hpf, embryos using *vmhc* (ventricle-specific marker gene) [28,29] and *amhc* (atrium-specific marker gene) [30,31] probes. The results indicated that *vmhc* was expressed on the left side of the midline in wild-type, *asb5a*^-*/*-^, and *asb5b*-MO embryos (viewed with the belly up position), while expression in the *asb5*-deficiency group was random, exhibiting three distinct patterns: Normal arrangement, Middle alignment, and Reverse arrangement (Figure 4A–F,A′–F′). Similarly, *amhc* was expressed on the right side of the midline in wild-type, *asb5a^-/-^*, and *asb5b*-MO embryos (also viewed belly up) with random expression observed in the *asb5*-deficiency group, which displayed the same three patterns: Normal arrangement, Middle alignment, and Reverse arrangement (Figure 4A″–F″,A′′′–F′′′). These findings suggest that the L-R asymmetry defect phenotype of the heart following the loss of *asb5* is not attributable to abnormal differentiation of the ventricular and atrial tissues, but rather to their abnormal positional arrangement.

### 2.4. Simultaneous Injection of asb5a and asb5b mRNA Can Effectively Rescue the Circularization Abnormal Phenotype in the asb5-Deficiency Group

**Figure 5:**
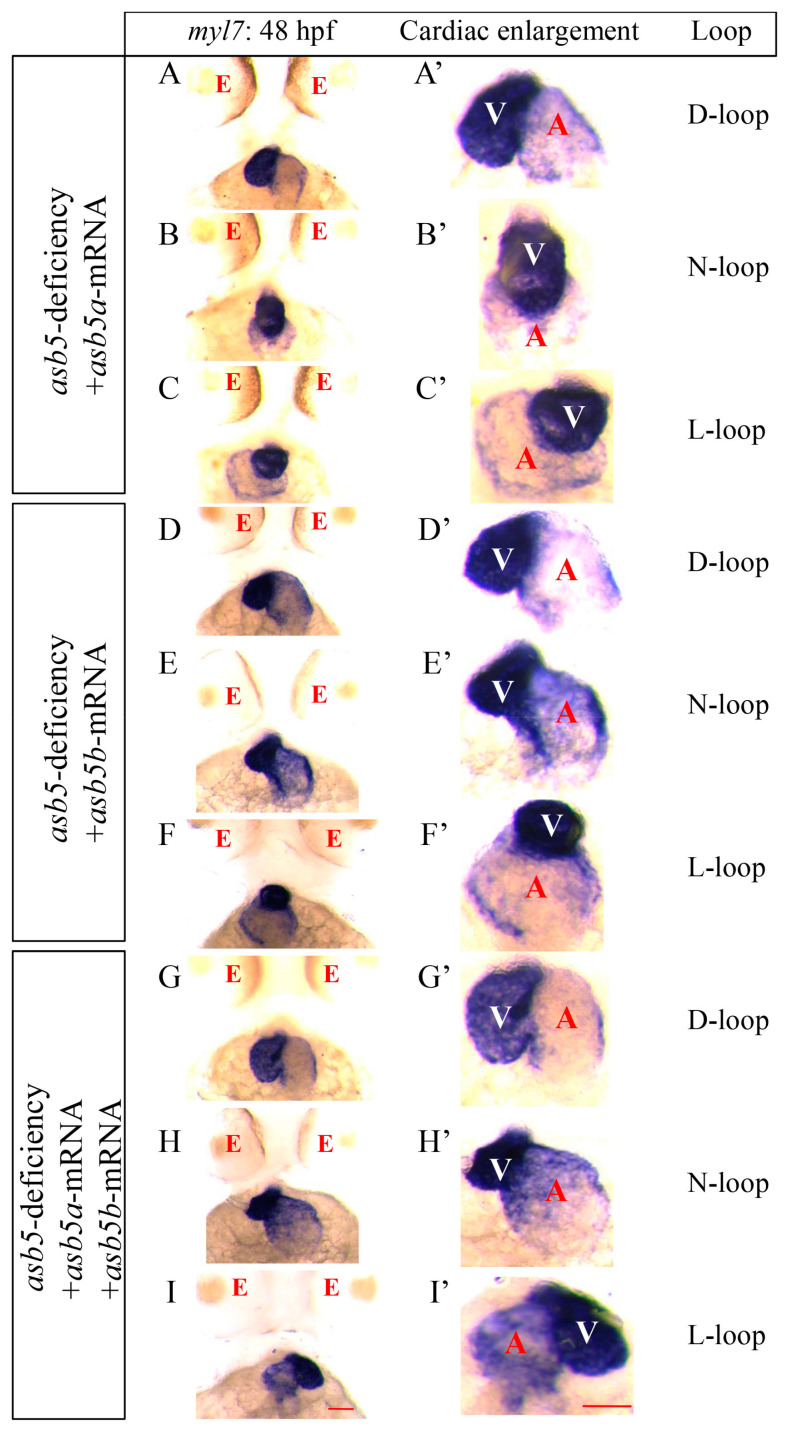
Rescue experiments conducted on the *asb5*-deficiency group marked by the *myl7* probe in embryos at 48 hpf. (**A**–**C**) Injection of *asb5a*-mRNA alone did not rescue the abnormal looping phenotype. (**D**–**F**) Injection of *asb5b*-mRNA alone also failed to rescue the abnormal phenotype. Images (**G**–**I**) show that the simultaneous injection of *asb5a* and *asb5b*-mRNA effectively rescued the phenotype in the *asb5*-deficiency group. Images (**A′**–**I′**) present magnified images of the heart, marked by the *myl7* probe, from various groups of embryos at 48 hpf. red E: eyes; white V: ventricle; red A: atrium; D-loop: dextral looping; N-loop: no looping; L-loop: levo-looping; Scale bar = 100 µm (belly up view).

**Figure 6:**
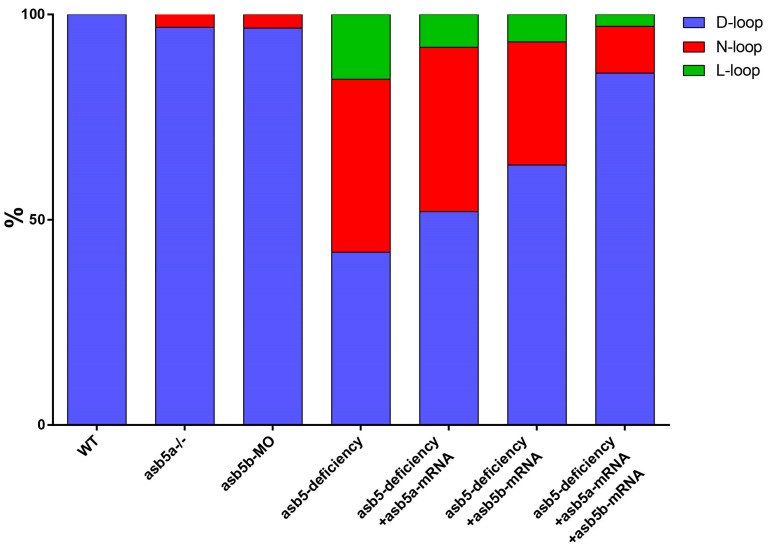
Statistical chart of rescue experiments conducted across various groups. WT: Wild-type control group. *asb5a*^-*/*-^: *asb5a* knockout homozygous group. *asb5b*-MO: *asb5b* knockdown group. *asb5-*deficiency: Simultaneous knockdown of *asb5b* group in *asb5a*^-*/*-^ homozygous embryos (loss of *asb5* group). *asb5-*deficiency + *asb5a*-mRNA: injection of *asb5a*-mRNA alone in the *asb5*-deficiency group. *asb5-*deficiency + *asb5b*-mRNA: injection of *asb5b*-mRNA alone in the *asb5*-deficiency group. *asb5-*deficiency + *asb5a*-mRNA + *asb5b*-mRNA: Simultaneous injection of *asb5a*-mRNA and *asb5b*-mRNA in the *asb5*-deficiency group (double rescue group). D-loop: dextral looping; N-loop: no looping; L-loop: levo-looping.

To eliminate the possibility that cardiac looping deformities were due to off-target effects or environmental factors, we conducted subsequent rescue experiments within the *asb5*-deficiency group. The results demonstrated that the injection of either *asb5a*-mRNA or *asb5b*-mRNA alone did not effectively rescue the asymmetric defect phenotype. In contrast, the simultaneous injection of *asb5a*-mRNA and *asb5b*-mRNA successfully rescued the L-R asymmetry defect in the heart (Figure 5). Statistical analyses confirmed that the circularization abnormalities in the *asb5*-deficient group were effectively rescued (Figure 6). These findings further substantiate that the early L-R asymmetry defects in the heart were indeed a consequence of the deletion of the *asb5* gene.

### 2.5. Loss of asb5 Affects the Expression of Genes Related to L-R Asymmetric Development

**Figure 7:**
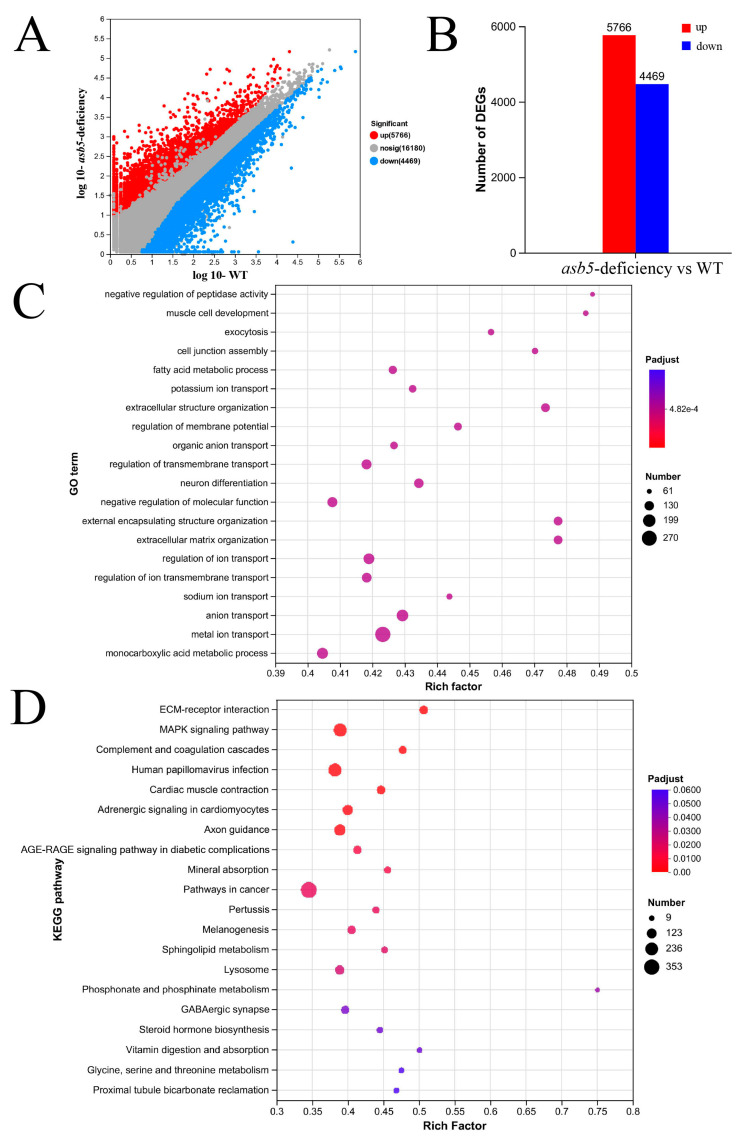
The impact of *asb5* loss on the transcriptome of 48 hpf *zebrafish* embryos. (**A**) The scatter plot displays expression differences for differentially expressed genes (DEGs), with blue dots indicating downregulated genes, red dots representing upregulated genes, and gray dots denoting genes with no significant expression differences across conditions (significance threshold: |log2FC| > 2.0 and *p* < 0.05). (**B**) The statistical chart summarizes expression differences for DEGs, where the red box highlights upregulated genes and the blue box indicates downregulated genes. (**C**) The bubble chart visualizes GO enrichment analysis for DEGs, with the vertical axis representing GO terms and the horizontal axis indicating the Rich factor, whereby a larger Rich factor signifies a higher degree of enrichment. The size of the bubbles corresponds to the number of genes/transcripts associated with each GO term, while the color of the dots corresponds to various ranges of Padjust. (**D**) The bubble chart depicts a KEGG pathway enrichment analysis for DEGs. The vertical axis lists the pathway names, while the horizontal axis indicates the Rich factor, where a larger Rich factor denotes a higher degree of enrichment. The size of the bubbles represents the number of genes in each pathway, and the color of the bubbles corresponds to varying ranges of Padjust.

To investigate the mechanism linking the *asb5* gene to L-R asymmetric development, we performed RNA-Seq high-throughput sequencing on 48 hpf *zebrafish* embryos from both the WT group and the *asb5*-deficient group (Figure 7). The results indicated that, compared to the WT group, the *asb5*-deficient group exhibited 5766 upregulated genes and 4469 downregulated genes (Figure 7A,B). Gene Ontology (GO) enrichment and Kyoto Encyclopedia of Genes and Genomes (KEGG) enrichment analyses highlighted the top 20 ranked entries (Figure 7C,D). Notably, the pathways related to extracellular matrix–receptor interaction, MAPK signaling, and adrenergic signaling in cardiomyocytes were closely associated with L-R asymmetric development. This finding aligns with the established role of structural ECM proteins and their integrin receptors in the development of the L-R axis of asymmetry in vertebrates [32]. Furthermore, our transcriptome results suggest that the hyperactivation of MAPK signaling mediated by Noonan syndrome and LEOPARD syndrome Shp2 variants may impair cilia function in Kupffer’s vesicle, leading to defects in L-R asymmetry and early cardiac development [33]. Additionally, adrenergic signaling facilitated by an increase in intracellular free calcium, may play a role in the asymmetric morphogenesis of visceral organs in Xenopus embryos [34].

**Figure 8:**
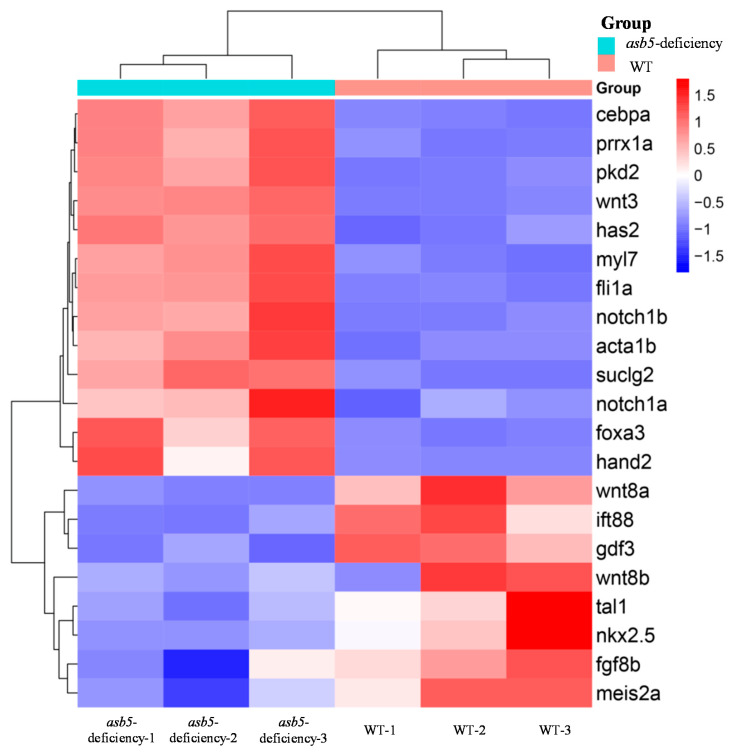
Summary chart of RNA-Seq data for the regulation of asymmetrically developing genes in 48 hpf *zebrafish* embryos. Heatmap of DEGs, where rows represent genes and columns represent samples. The red and blue colors indicate high and low expression levels, respectively, with darker colors denoting more pronounced significant differences.

**Figure 9:**
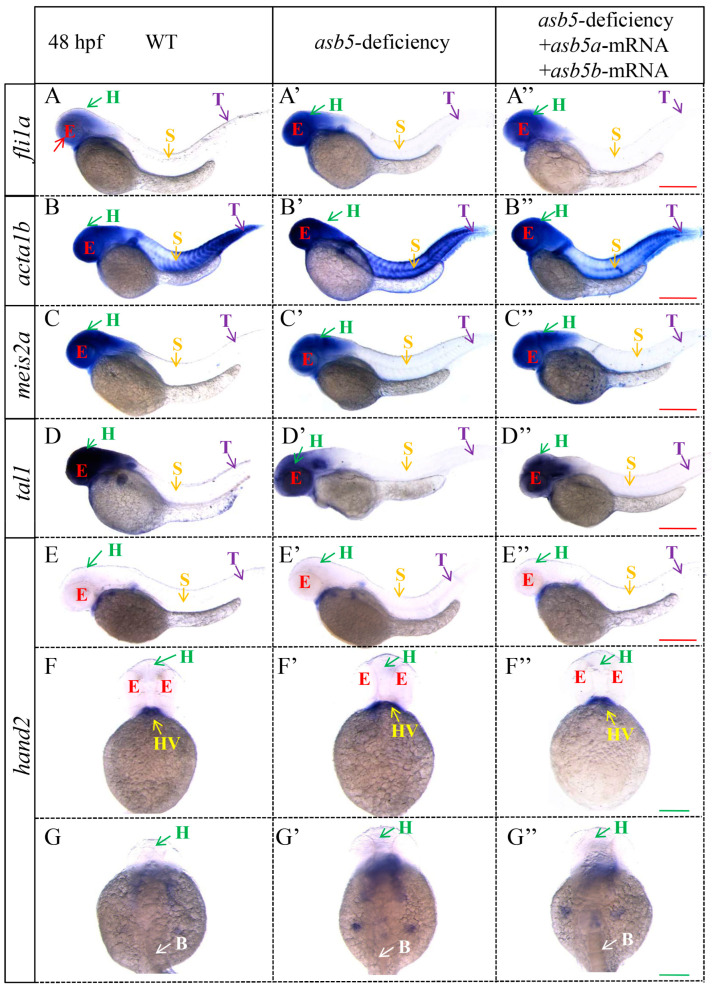
WISH results for genes associated with L-R asymmetric development in 48 hpf *zebrafish* embryos. Images (**A**–**A″**) illustrate the WISH expression of *fli1a* in WT, *asb5-*deficient, and *asb5-*deficient +*asb5a*/*asb5b*-mRNA groups (side view). Images (**B**–**B″**) display the WISH expression of *acta1b* across the different groups (side view). Images (**C**–**C″**) show the WISH expression of *meis2a* in various groups (side view). Images (**D**–**D″**) present the WISH expression of *tal1* in different groups (side view). Images (**E**–**E″**) display the WISH expression of *hand2* across various groups (side view). Images (**F**–**F″**) depict the WISH expression of *hand2* in the multiple groups (abdominal view). Images (**G**–**G″**) show the WISH expression of *hand2* in different groups (back view). red E: eyes; green H: head; orange S: skeletal muscle; purple T: tail; yellow HV: heart valves; white B: back; Red scale bar = 500 µm; Green scale bar = 250 µm.

**Figure 10:**
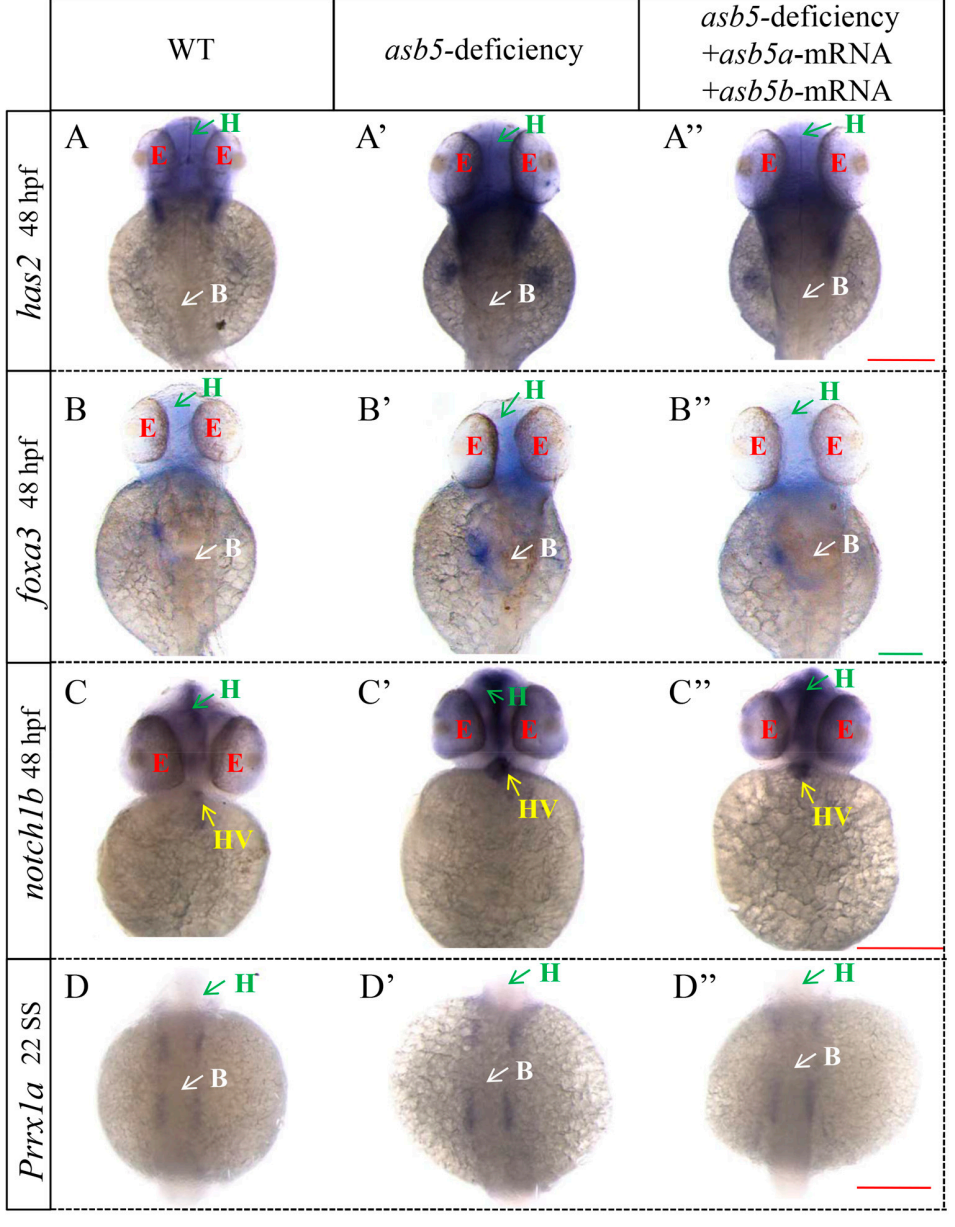
WISH results for other genes related to L-R asymmetric development in *zebrafish* embryos at 48 hpf across various groups. Images (**A**–**A″**) illustrate the WISH expression of *has2* in WT, *asb5-*deficiency group, and *asb5-*deficiency group + *asb5a*/*asb5b*-mRNA group (back view). Images (**B**–**B″**) show the WISH expression of *foxa3* across different groups (back view). Images (**C**–**C″**) depict the WISH expression of *notch1b* in various groups (abdominal view). Images (**D**–**D″**) present the WISH expression of *prrx1a* in the same groups (abdominal view). 22 SS: 22 somite stage; green H: head; white B: back; red E: eyes; yellow HV: heart valves; Red scale bar = 500 µm; Green scale bar = 250 µm.

A further analysis of the RNA-seq data revealed that, compared to the WT group, the *asb5*-deficiency group exhibited an imbalance in the expression of numerous genes associated with L-R asymmetric development (Figure 8, Supplementary File S2). Notably, the expressions of *cebpa* [35,36], *prrx1a* [2,37], *pkd2* [38,39], *wnt3* [40], *has2* [41], *myl7*, *fli1a* [42,43], *notch1b* [44,45], *acta1b* [46,47], *suclg2* [48], *notch1a* [49], *foxa3* [50,51], and *hand2* [52] were significantly upregulated (Figure 8, Red). In contrast, the expressions of *wnt8a* [53], *ift88* [54,55], *gdf3* [56,57], *wnt8b* [58], *tal1* [59,60], *nkx2.5* [61,62], *fgf8b* [63], and *meis2a* [64] were significantly downregulated (Figure 8, Blue). WISH was subsequently conducted, confirming that *fli1a*, *acta1b*, *hand2*, *has2*, *prrx1a*, *notch1b,* and *foxa3* were significantly upregulated, while *meis2a* and *tal1* were significantly downregulated (Figure 9 and Figure 10).

### 2.6. Nodal Signaling-Related Genes Are Expressed Imbalanced in the asb5-Deficiency Group

**Figure 11:**
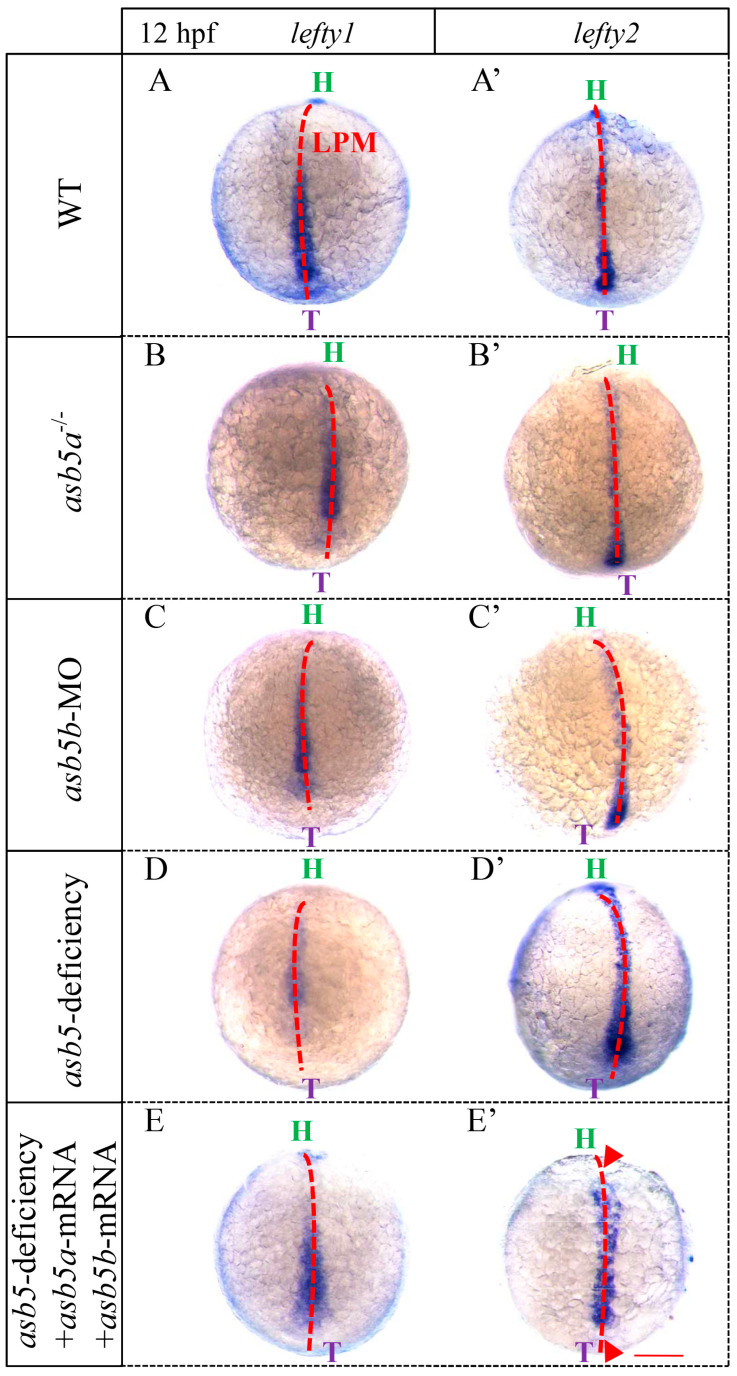
WISH results utilizing *lefty1* and *lefty2* probes in embryos at 12 hpf across various groups. Images (**A**–**E**) depict schematic diagrams illustrating *lefty1* expression in the LPM of the embryos. Images (**A′**–**E′**) show corresponding schematic diagrams for *lefty2* expression in the LPM of the embryos. green H: head; purple T: tail; LPM: lateral plate mesoderm; red triangular arrow: indicates the region where *lefty2* expression is downregulated following double rescue; Scale bar = 500 µm (back up view).

**Figure 12:**
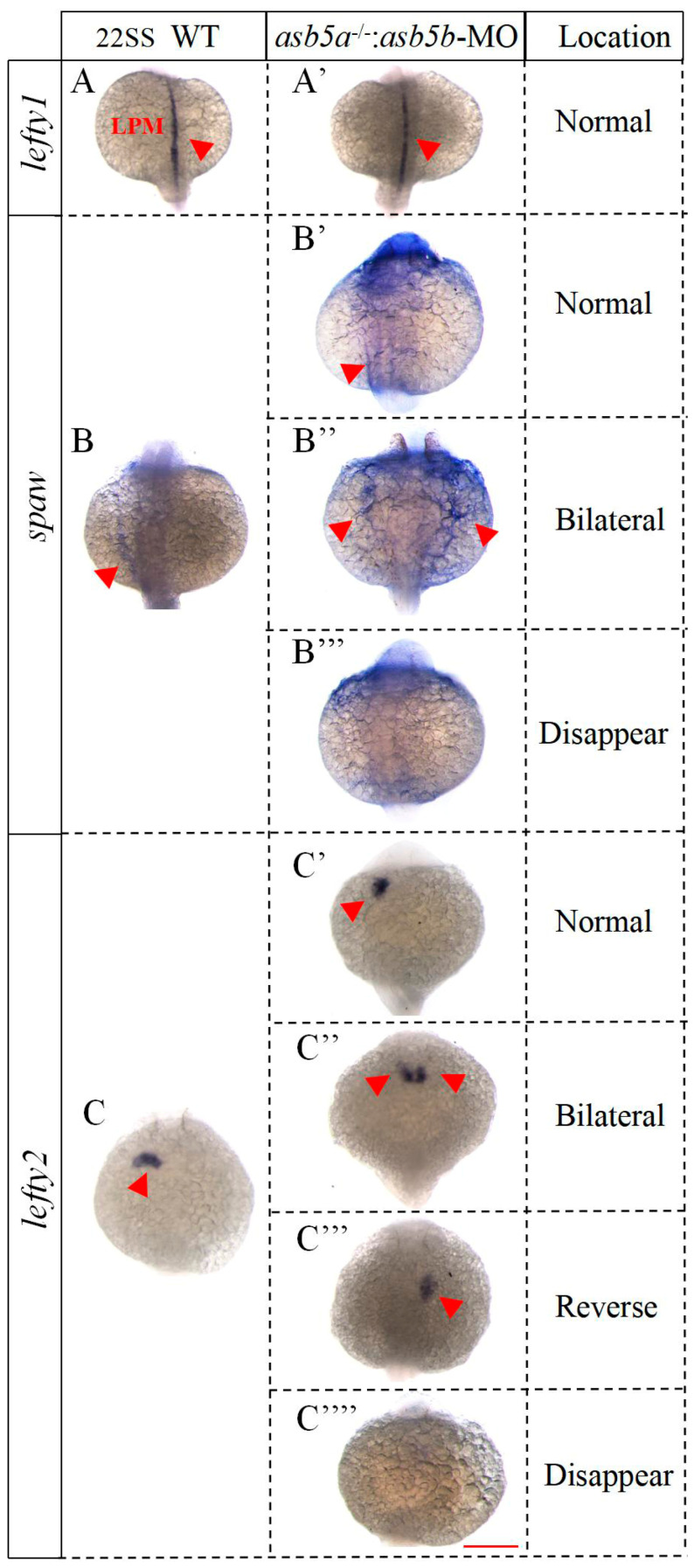
WISH results in WT and the *asb5*-deficiency group *zebrafish* embryos at 22 SS. (**A**–**A′**) The schematic diagram illustrates the expression of *lefty1* in the LPM of the embryos. (**B**–**B′′′**) This diagram depicts *spaw* expression in proximity to the LPM. In the WT group, *spaw* is localized on the left side of the LPM, whereas the *asb5*-deficiency group displays three distinct expression patterns: left-side expression, bilateral expression, and absence of expression. (**C**–**C′′′′**) The schematic diagram shows *lefty2* expression near the LPM in embryos. In the WT group, *lefty2* is expressed on the left side of the LPM, while the *asb5*-deficiency group reveals four expression patterns: left-side expression, bilateral expression, reverse expression, and no expression. LPM: lateral plate mesoderm; Red triangular arrow: positive expression region; Scale bar = 500 µm (back up view).

The above results demonstrated that at 48 hpf during *zebrafish* embryonic development, the *asb5*-deficiency group displayed abnormal cardiac L-R asymmetry, accompanied by an imbalance in the expression of genes associated with asymmetric development. Subsequently, we aimed to investigate whether this abnormality is linked to the Nodal signaling pathway, which plays a crucial role in the development of L-R asymmetry at earlier stages. We noted that in *zebrafish*, the Nodal protein homolog gene is *spaw*, expressed laterally on the left LMP. *spaw* subsequently regulates the expression of downstream target genes *lefty1* and *lefty2*, which act as antagonists to *spaw* and can also exert negative feedback to modulate *spaw* expression [7,11,12]. Consequently, we conducted WISH experiments to assess the expression of these genes at earlier stages. The results revealed that *lefty1* and *lefty2* were specifically expressed in the LPM of the embryonic dorsal region in WT *zebrafish* at 12 hpf embryonic development. In contrast, *lefty1* expression in the *asb5*-deficiency group was significantly downregulated, while *lefty2* was significantly upregulated. Notably, this altered expression pattern was effectively rescued by the co-injection of *asb5a*-mRNA and *asb5b*-mRNA (Figure 11). At the 22 somite stage (SS) of embryonic development, *lefty1* continued to be expressed at the midline in the *asb5*-deficiency group, showing no significant differences compared to the WT group (Figure 12A–A′). Typically, *spaw* is expressed in the LPM with a left-sided bias (Figure 12B); however, in the *asb5*-deficiency group, three distinct expression patterns were observed: Normal, Bilateral, and Disappeared expression (Figure 12B′–B′′′). In the WT group at the 22 SS, *lefty2* is specifically expressed on the left side of the LPM (Figure 12C). In contrast, the *asb5*-deficiency group exhibited four expression patterns: Normal, Bilateral, Reverse, and Disappeared expression (Figure 12C′–C′′′′). In summary, following the inactivation of the two subtypes of *asb5* in *zebrafish*, the Nodal homolog gene *spaw* demonstrated random expression or the absence of expression, while its downstream target gene *lefty1* was downregulated and *lefty2* was upregulated, also demonstrating random distribution (Figure S6). These findings suggest that the two isoforms of *asb5*, *asb5a* and *asb5b*, may regulate the expression of *spaw*, *lefty1*, and *lefty2* via the Nodal signaling pathway, which is essential for establishing the early cardiac asymmetry pattern in *zebrafish*.

### 2.7. Construction of the PPI (Protein–Protein Interaction) Regulatory Network Diagram

**Figure 13:**
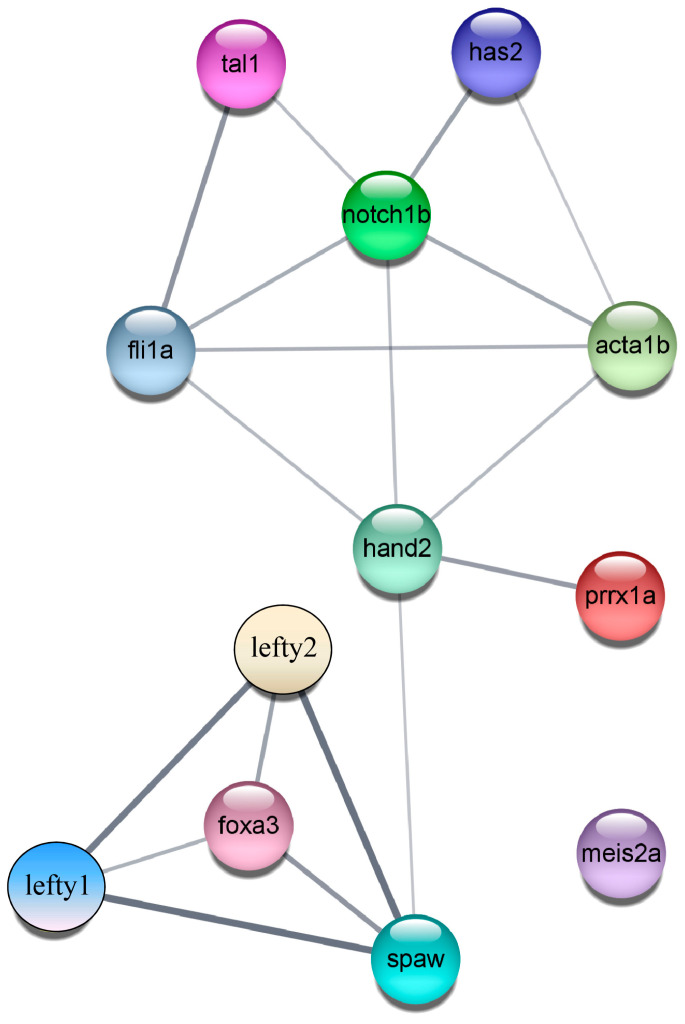
PPI regulatory network diagram. The PPI network comprises three modules, consisting of a total of 12 nodes and 18 edges.

Based on the results presented, we found that under normal circumstances, the Nodal gene *spaw* is expressed in the left LPM and activates the Nodal signaling pathway on the left side of the heart [8]. However, following the inactivation of both the *asb5a* and *asb5b* genes, the Nodal signaling pathway associated with L-R asymmetric development is altered. The Nodal protein homolog gene *spaw* begins to exhibit random expression, leading to a significant downregulation of its downstream target gene *lefty*. *Lefty1* acts as a `molecular midline barrier’ at the midline, preventing Nodal propagation from the left to the right LPM [10]. Subsequently, the expression of the laterality gene *lefty2* becomes unbalanced, resulting in the dysregulation of a series of genes linked to L-R asymmetric development. To investigate this further, we utilized the STRING database (version 11.0b) [65,66] and Cytoscape software (version 3.9.1) [67,68] to construct a unified regulatory PPI network diagram of these 12 differentially expressed genes. Our analysis revealed that the regulation of these genes can be broadly categorized into three modules: First, following the loss of *asb5*, the earliest laterality gene *spaw* exhibits unbalanced expression, resulting in a significant downregulation of the downstream gene *lefty1*. The absence of *lefty1* disrupts the midline barrier, allowing the left-sided specific factor *lefty2* to cross the midline and express randomly. Consequently, the proper pattern of the early lateral Nodal signaling pathway is disrupted, affecting the correct expression of *foxa3*, which is a specific marker for the liver, intestine, and pancreas [50,51]. Secondly, the loss of *asb5* leads to an imbalance in the expression of left-sided *spaw* and right-sided *prrx1a*, which impacts the expression of the early cardiac marker gene *hand2*. This disruption results in an imbalance in the expression of genes associated with cardiac valve development, vascular endothelial formation, and skeletal muscle development, including *fli1a, tal1*, *has2*, *notch1b*, and *acta1b*. Furthermore, the downregulation of *meis2a* following the loss of *asb5* may directly influence the establishment of the heart’s L-R asymmetry pattern (Figure 13).

## 3. Discussion

Previous research conducted by our team demonstrated that the application of CRISPR/Cas9 and morpholino techniques to inactivate the expression of *asb5* in *zebrafish* (*asb5*-deficiency group) led to a phenotype characterized by abnormal cardiac contraction. This finding underscores the critical role of *asb5* in sustaining normal heart function during development. In this study, we observed cardiac looping abnormalities at 48 hpf in the *asb5*-deficiency group, providing the first evidence that the *asb5* gene is significant for maintaining the L-R asymmetrical development of the early heart in *zebrafish*.

Initially, we utilized the *myl7*:EGFP *zebrafish* line as a background to observe cardiac looping abnormalities at 48 hpf in the *asb5*-deficiency group. This observation was subsequently confirmed through analysis using the Semi-automatic Heartbeat Analysis software program (Version 3.4.0.0). To eliminate the possibility that these deformities arose from environmental factors (such as water quality, food, and pH), we repeated the experiments and randomly collected 48 hpf embryos from various groups for WISH. The results indicated that the *asb5*-deficiency group exhibited three distinct cardiac looping phenotypes—D-loop, N-loop, and L-loop—when assessed with the *myl7* and *nppa* probes, in comparison to the other groups. This finding confirmed that the absence of *asb5* in *zebrafish* indeed resulted in abnormalities in early cardiac looping. In situ hybridization results utilizing *vmhc* and *amhc* probes demonstrated that the differentiation of ventricular and atrial tissues was normal; however, their positions displayed random distributions (Normal arrangement, Middle alignment, and Reverse arrangement). Furthermore, rescue experiments were conducted, revealing that only the simultaneous injection of *asb5a* and *asb5b* mRNA effectively rescued the cardiac looping abnormality phenotype in the *asb5*-deficiency group. In summary, the deletion of *asb5* in *zebrafish* results in abnormalities in the early L-R asymmetrical development of the heart. Subsequently, RNA-Seq results revealed an expression imbalance in a series of genes associated with L-R asymmetrical development in the *asb5*-deficiency group. WISH experiments confirmed the expression imbalance of 12 genes (*fli1a*, *acta1b*, *hand2*, *has2*, *prrx1a*, *notch1b*, *foxa3*, *meis2a*, and *tal1)*. We then investigated whether this gene expression imbalance was closely related to the early regulatory Nodal signaling pathway that governs L-R asymmetrical development. The expression levels of classic marker genes for the Nodal signaling pathway *spaw*, *lefty1*, and *lefty2* were assessed. The WISH results indicated that these three genes exhibited abnormal expression following the deletion of *asb5*.

*Meis2a* is expressed in the gill arches of *zebrafish* and exhibits a similar expression pattern to *meis2b*. The literature indicates that *meis2b* mutant *zebrafish* display cardiac circulation defects and a reduced heart rate.

Spaw [69] is recognized as the earliest confirmed nodal homologous gene associated with L-R asymmetry, typically expressed in the left LPM. It subsequently regulates the expression of its downstream target genes, *lefty1* and *lefty2*. The Nodal signaling pathway is highly conserved on the left side during embryonic development, and its proper regulation is essential for L-R asymmetric development. *Lefty1* is expressed at the midline, functioning as a “molecular midline barrier” that prevents the propagation of Nodal from the left to the right LPM [10]. The downregulation of *lefty1* disrupts this midline barrier, enabling the expression of the laterality gene *lefty2* to cross the midline and manifest in the right LPM. *Foxa3* [70,71] serves as a marker gene for the liver, intestine, and pancreas. Alterations in the upstream Nodal signaling pathway can result in the mislocalization of visceral organs and may also influence the expression of *foxa3*. The *prrx1a* gene in *zebrafish* [72,73] exhibits stronger expression in the right LPM compared to the left. The *bmp4*-*prrx1a* signaling axis plays a critical role in regulating L-R asymmetric development on the right side of the midline.

Additionally, *hand2* is crucial for heart development, with its expression primarily localized to the right side of the heart tube. And *zebrafish* atrioventricular valve formation necessitates the transition from epithelial to mesenchymal cells. The Notch signaling pathway, BMP signaling pathway, and Wnt signaling pathway collaborate to facilitate the EMT, with genes such as *notch1b*, *bmp4,* and *has2* playing critical roles in this process [74,75]. *Tal1* has been identified as a key factor in early endothelial growth, regulating the formation of intercellular junctions and the maintenance of endocardial characteristics [76]. *Fli1a* is recognized as a marker gene for vascular development in *zebrafish* [31,32], while *acta1b* is involved in regulating skeletal muscle development in *zebrafish*. The changes in the abovementioned genes may have affected the normal development of blood vessels, endocardium, and valves in the heart tissue, ultimately resulting in the abnormal L-R asymmetry of the heart. 

**Figure 14:**
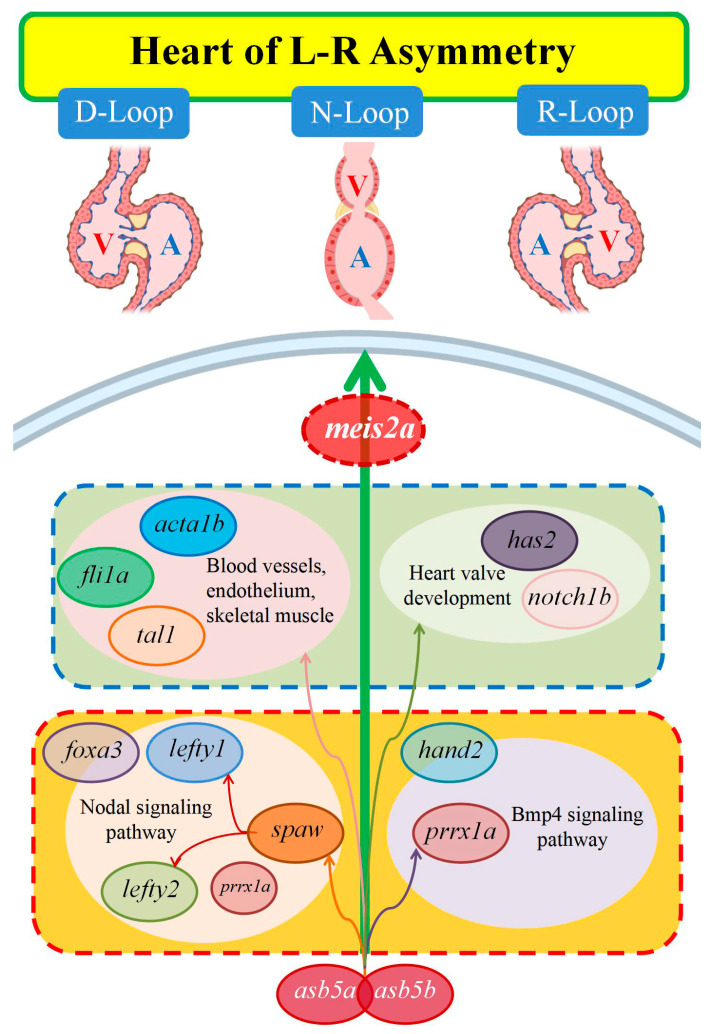
This molecular regulatory network diagram illustrates the influence of *asb5a*/*asb5b* on the L-R asymmetric development of the heart in *zebrafish*. V: ventricle; A: atrium; D-loop: dextral looping; N-loop: no looping; L-loop: levo-looping.

Through an analysis of the preliminary PPI network depicted in Figure 13 and a review of the existing literature, we constructed a probable molecular regulatory network influencing early cardiac L-R asymmetry in *zebrafish* (Figure 14). We hypothesize that *asb5* deficiency may impact the early cardiac L-R asymmetric development in *zebrafish* through three primary pathways: First, the loss of *asb5* leads to the dysregulation of *meis2a* expression, which may directly contribute to the early L-R asymmetric development of the heart. Second, *asb5* deficiency disrupts *spaw* expression, resulting in significant downregulation of the downstream target gene *lefty1*, thereby compromising the midline barrier. Consequently, the expression of *spaw* and *lefty2* on the left side crosses the midline barrier to the right side of the LPM, ultimately leading to errors in the conserved Nodal signaling pathway on the left side and the dysregulation of *foxa3*. Finally, the absence of *asb5* results in an imbalance in the expression of *prrx1a* on the right side, a gene that is typically expressed at low levels on the left side and at high levels on the right side, where it participates in the *bmp4*-*prrx1a* signaling pathway. Additionally, *hand2* is highly expressed on the right side during embryonic development, potentially co-regulating right-sided Bmp4 signaling to facilitate L-R asymmetric development. The disruption of these L-R signaling pathways may lead to an imbalance in the expression of a series of genes involved in heart valve development, vascular endothelial development and skeletal muscle development, ultimately severely affecting the early asymmetric development of the heart (Figure 14).

In summary, our study has preliminarily confirmed the critical role of the *asb5* gene in the early L-R asymmetric development of the heart in *zebrafish*. The deficiency of *asb5* disrupts the expression of genes associated with the L-R axis, thereby affecting the morphogenesis of early cardiac looping. This finding offers significant insights for further research into the molecular regulatory mechanisms of this gene in heart development and enhances the understanding of potential therapeutic targets for early congenital heart disease.

## 4. Materials and Methods

### 4.1. Zebrafish Strain Rearing and Breeding

The AB *zebrafish* strain and the *myl7*:EGFP strain, which specifically expresses green fluorescent protein in cardiac tissue, were maintained at a constant temperature of 28.5 °C in a water circulation system. For *zebrafish* breeding, two females and three males were placed into hybrid bars, with clear plastic partitions used for overnight separation. The partitions were removed around 09:00 am the following day to facilitate mating, egg laying, and subsequent embryo collection.

### 4.2. Imaging Technique and Image Analysis

An Axiocam from Zeiss Company was utilized to capture and analyze the hearts of *myl7*:EGFP *zebrafish* at 48 hpf. To minimize *zebrafish* movement, a 6% methylcellulose solution was employed. Image processing was performed using the Zeiss AxioVision 3.0.6 software package ((Zeiss Company, Jena, Germany) in conjunction with Adobe Photoshop. A high-speed EM-CCD camera recorded the *zebrafish* heartbeats at a rate of 130 frames per second for a duration of 10 s, utilizing a 20× objective lens. Subsequently, the recorded heartbeat movie was analyzed with the Semi-automatic Heartbeat Analysis software program. Each frame of the movie, with a resolution of 1300 × 600 pixels, was extracted to examine the cardiac looping morphology during early development. Fluorescence imaging of WISH-stained *zebrafish* embryos was conducted using a Leica M205FA microscope (Wetzlar, Germany), with a 10% methylcellulose solution employed to immobilize the stained embryos. All in situ hybridization images were captured using the Leica Application Kit imaging software program (version 3.2.0).

### 4.3. Synthesis of RNA Probes and WISH in Embryos

To prepare the mRNA antisense probe, a segment of the mRNA sequence corresponding to the gene was amplified using reverse transcription polymerase chain reaction (RT-PCR). The forward and reverse primers, which include the T7 promoter sequence, are detailed in Table S1. The purified RT-PCR products served as the transcription template for synthesizing digoxigenin-labeled antisense RNA probes via in vitro transcription, utilizing the Riboprobe^®^ System-T7 transcription kit (P1440, Promega, Madison, WI, USA) and the ROACH DIG RNA Labeling Mix (REF 11277073910, Roche, Basel, Switzerland). *Zebrafish* embryos at specific developmental stages were fixed overnight in 4% paraformaldehyde and subsequently stored in 100% methanol. WISH was employed to detect the spatial distribution and expression levels of the gene.

### 4.4. In Vitro Synthesis of Cap-Modified mRNA for asb5a and asb5b

To prepare cap-modified mRNA, the complete coding sequence (CDS) of the gene was amplified using RT-PCR. The forward and reverse primers, which include the T7 promoter sequence, are detailed in Table S2. The purified RT-PCR products were ligated into the pXT7 empty vector to construct an overexpression plasmid (Figures S1–S3). Following single enzyme digestion of the overexpression plasmids, they served as the transcription template for synthesizing cap-modified, active mRNA via in vitro transcription, utilizing the Riboprobe^®^ System-T7 transcription kit (P1440, Promega, Madison, WI, USA) and Ribo m7G Cap Analog (P1712, Promega, Madison, WI, USA).

### 4.5. RNA-Seq Transcriptome Analysis

Collect 48 hpf *zebrafish* embryos, with 50 embryos per tube constituting one biological replicate, and store them at −80 °C. Each dataset in the RNA-seq analysis is derived from a biological replicate consisting of 50 embryos. The RNA-seq sequencing was performed by Majorbio, and the RNA-seq data analysis was conducted using the free online Majorbio Cloud platform (www.majorbio.com). DEGs were analyzed using DESeq, with the screening criteria established as follows: |log_2_ fold change| > 2.0 and a significance *p*-value < 0.05.

### 4.6. Construction of PPI Network and Module Screening

The PPI network was constructed using STRING version 11.0b (available at https://string-db.org/, accessed on 2 June 2022) in conjunction with Cytoscape (version 3.7.1).

### 4.7. Statistical Analyses

The results are the mean ± SEM. Data were analyzed using Student’s *t*-test or a one-way ANOVA. Significance was defined as *p* < 0.05.

## 5. Conclusions

In conclusion, our results indicate that the *asb5* gene plays an important role in the early heart development of *zebrafish* and is crucial for the normal maintenance of the L-R asymmetric development of the heart, providing new targets and directions for the clinical diagnosis and treatment of *zebrafish* heart diseases.

## Data Availability

All data generated or analyzed during this study are included in the article/Supplementary Material. Further inquiries can be directed to the corresponding authors.

## Supplementary Materials

The following supporting information can be downloaded at: https://www.mdpi.com/article/10.3390/ijms26062765/s1.

## Abbreviations

The following abbreviations are used in this manuscript

MO	Morpholino
Hpf	Hours post fertilization
WT	Wild type
WISH	Whole-mount in situ hybridization
LPM	Lateral plate mesoderm
L-R	Left–right
D-loop	Dextro-looping
N-loop	No looping
L-loop	Levo-looping
RT-PCR	Reverse transcription polymerase chain reaction
CDS	Coding sequence
DEGs	Differentially expressed genes
PPI	Protein–protein interaction
GO	Gene Ontology
KEGG	Kyoto Encyclopedia of Genes and Genomes
SS	Somite stage
