# Supplementary material for: The Interaction Between the asb5a and asb5b Subtypes Jointly Regulates the L-R Asymmetrical Development of the Heart in Zebrafish
Source: Int J Mol Sci. 2025 Mar 19;26(6):2765. doi: 10.3390/ijms26062765 (PMC11943173; doi:10.3390/ijms26062765)
Supplement: Supplementary file 1 [file ijms-26-02765-s001.zip › proofreading-Supplementary Flie S1.pdf]

Table S1: Primers sequences used for WISH

Primer	Sequences (5′→3′)
myl7–WISH-F	ggctcttccaatgtcttc
myl7–WISH-T7R	CCCTAATACGACTCACTATAGGGtatttccagccacgtcta
vmhc–WISH-F	attctgaggtggcacagtgg
vmhc–WISH-T7R	CCCTAATACGACTCACTATAGGGctgcagaaggttgttgcgtc
amhc–WISH-F	gatccgacaaactcctgaaaac
amhc–WISH-T7R	CCCTAATACGACTCACTATAGGGgtgtttccgtaatgcatgatagc
fli1a–WISH-F	ggatccagagagtcgccggt
fli1a–WISH-T7R	CCCTAATACGACTCACTATAGGG aggacctcggtgttataaac
acta1b–WISH-F	atgtgtgacgacgacgagac
acta1b–WISH-T7R	CCCTAATACGACTCACTATAGG gctcagtcaagatcttcatg
meis2a–WISH-F	ctcgaaaaagggggcatctt
meis2a–WISH-T7R	CCCTAATACGACTCACTATAGGG caattctggattcgaagaaa
tal1–WISH-F	gatataaaaggcagggagct
tal1–WISH-T7R	CCCTAATACGACTCACTATAGG gagcttcaccgccgaccatg
has2–WISH-F	agaggacccgaagaaactga
has2–WISH-T7R	CCCTAATACGACTCACTATAGG gtctgatcataccagtcctc

Table S2: Primer sequences for constructing overexpression plasmids

Primer	Sequences (5′→3′)
asb5a-CDS-F	CAACTTTGGCAGATCGGTACCatgacagagtccacagagga
asb5a-CDS-R	AGTGATATCAGATCTCTCGAGttatctgtattgtagaaagt
asb5b–CDS-F	CAACTTTGGCAGATCGGTACCatgactgagattttagagga
asb5b–CDS-R	AGTGATATCAGATCTCTCGAGttatctgtattgaaggaagc

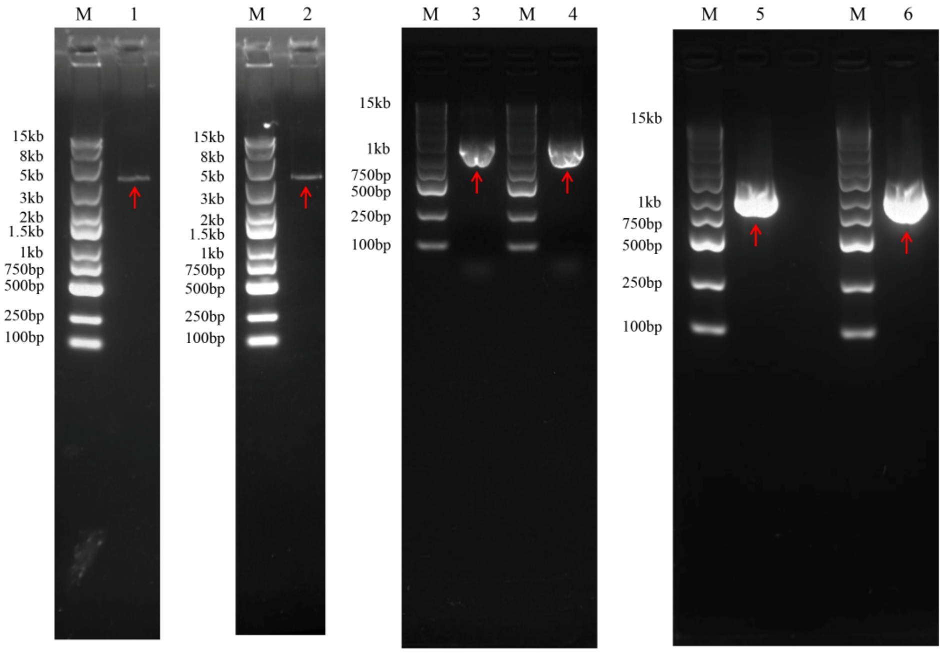

Figure S1. The electrophoresis maps for the construction of the *asb5a*/*asb5b* overexpression plasmids. Lane 1: the empty vector pXT7 following double enzyme digestion with Kpn I and Xho I. Lane 2: the purified double enzyme digestion product of pXT7 DNA. Lane 3: the PCR product for the *asb5a*- coding sequence (CDS). Lane 4: the PCR product for the *asb5b*-CDS. Lane 5: the purified PCR product for *asb5a*-CDS. Lane 6: the purification recovery of the PCR product for *asb5b*-CDS. Lane M: DNA marker (15 kb). Red arrow: the positive band.

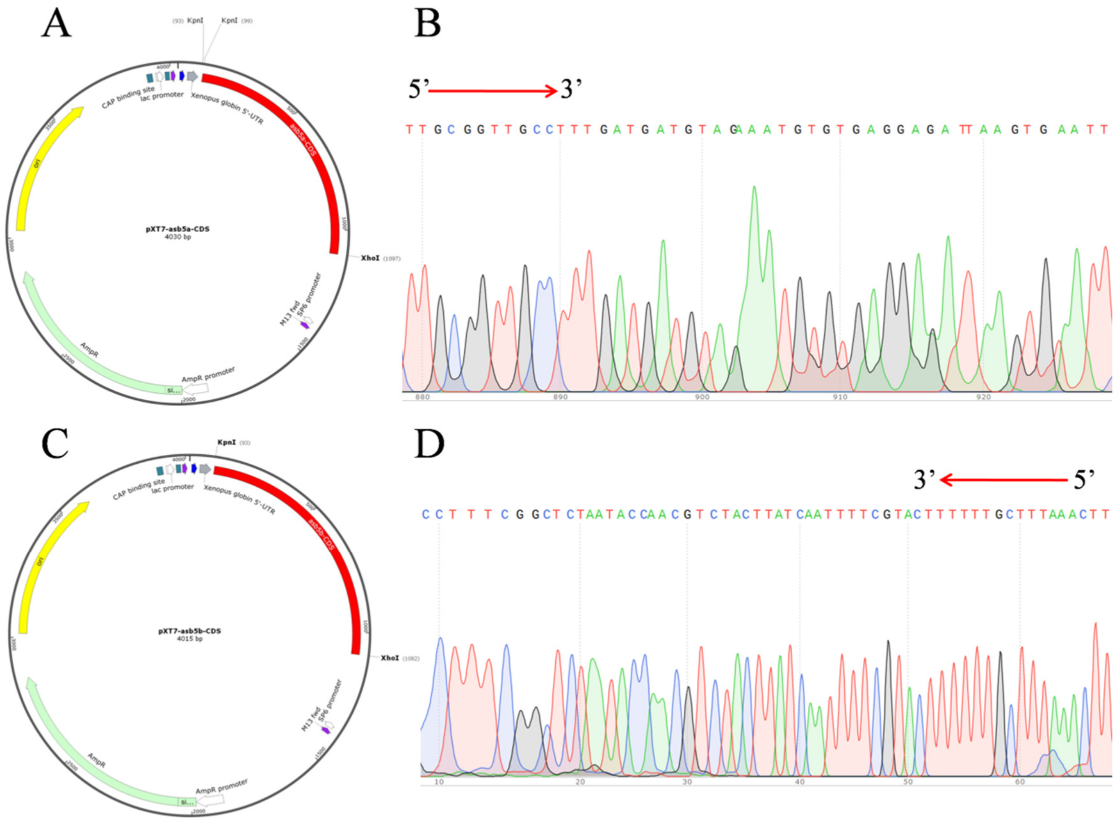

Figure S2. Schematic diagrams of overexpression plasmids construction and sequencing peaks. A/C: Schematic diagram of constructing overexpression plasmids of *asb5a* and *asb5b*,

B/D: Overexpression plasmid Sanger sequencing peak diagram, in which bases A, G, C, and T are represented by green, black, blue, and red curves, respectively.

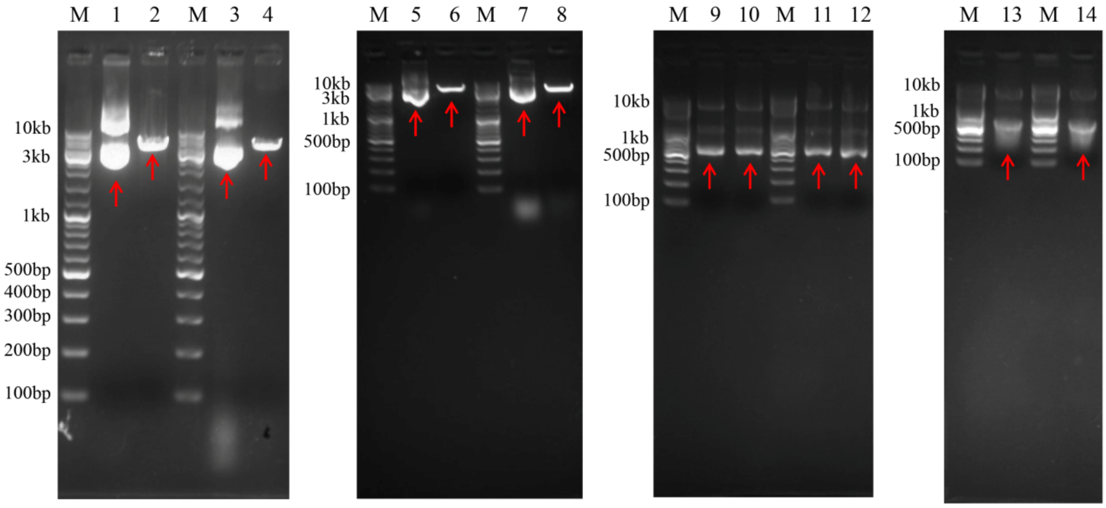

Figure S3. The electrophoresis maps of synthesized the *asb5a*/*asb5b* capped mRNA. Lane 1/5: pXT7-*asb5a*-CDS overexpression plasmid. Lane 2: pXT7-*asb5a*-CDS overexpression plasmid product cleaved by the Xho I enzyme. Lane 3/7: pXT7-*asb5b*-CDS overexpression plasmid. Lane 4: pXT7-*asb5b*-CDS overexpression plasmid product cleaved by Xho I. Lane 6/8: purification and recovery the enzyme digestion product of the pXT7-*asb5a*-CDS/pXT7-*asb5b*-CDS overexpression plasmid. Lanes 9/10: T7 in vitro cap transcription *asb5a*-mRNA. Lanes 11/12: T7 in vitro cap transcription *asb5b*-mRNA. Lane 13/14: the purification recovery of the *asb5a*-mRNA/*asb5b*-mRNA. Lane M: DNA Marker (10 kb). Red arrow: positive band.

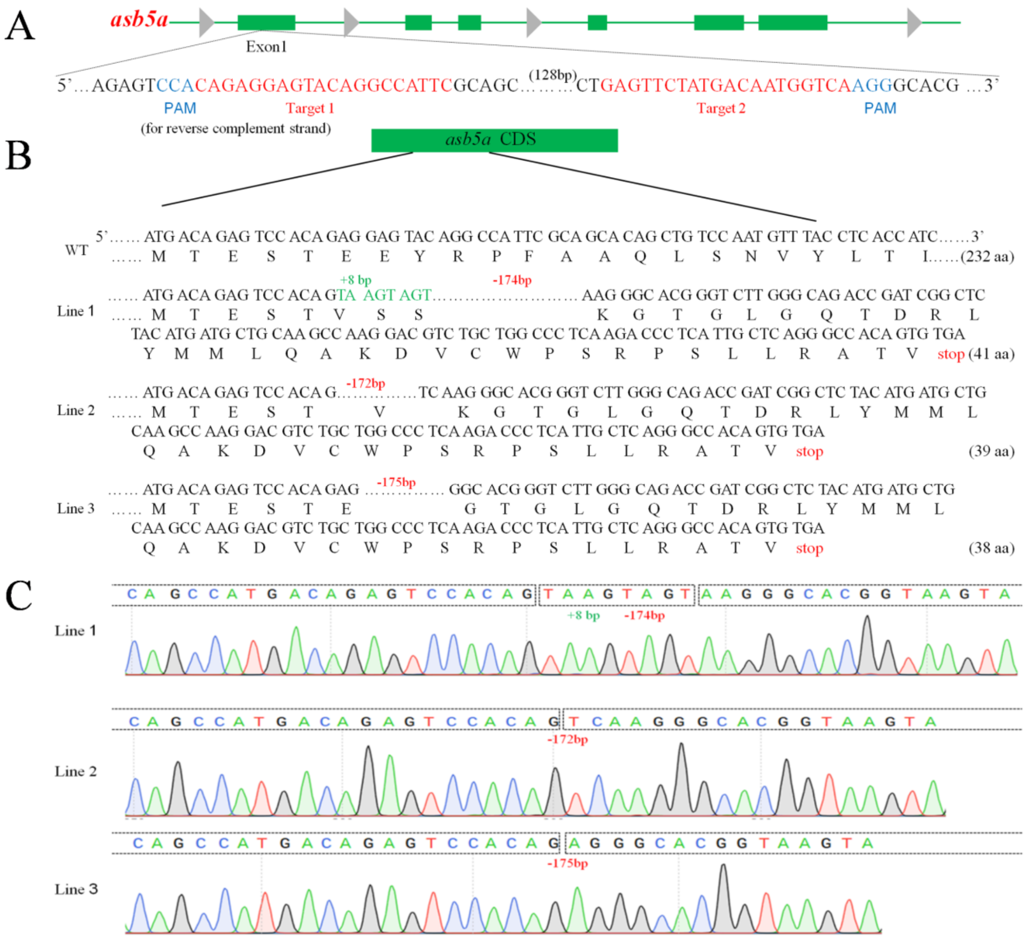

(International Journal of Molecular Sciences, 2023, 16364)

Figure S4. Schematic diagrams of *asb5a* gene knockout in zebrafish. (A) Schematic diagram of sgRNA targeting for *asb5a* gene knockout. Green horizontal line represents the genomic DNA of *asb5a*, green rectangle represents the exons of *asb5a*, red font represents the target site sequence, and blue font represents the protospacer adjacent motif (PAM). (B) Schematic diagram of the three heritable mutant alleles of *asb5a* produced via gene knockout and their encoded protein sequences. (C) Sequencing peak diagram of three *asb5a* mutant alleles, in which bases A, G, C, and T are represented by green, black, blue, and red curves, respectively.

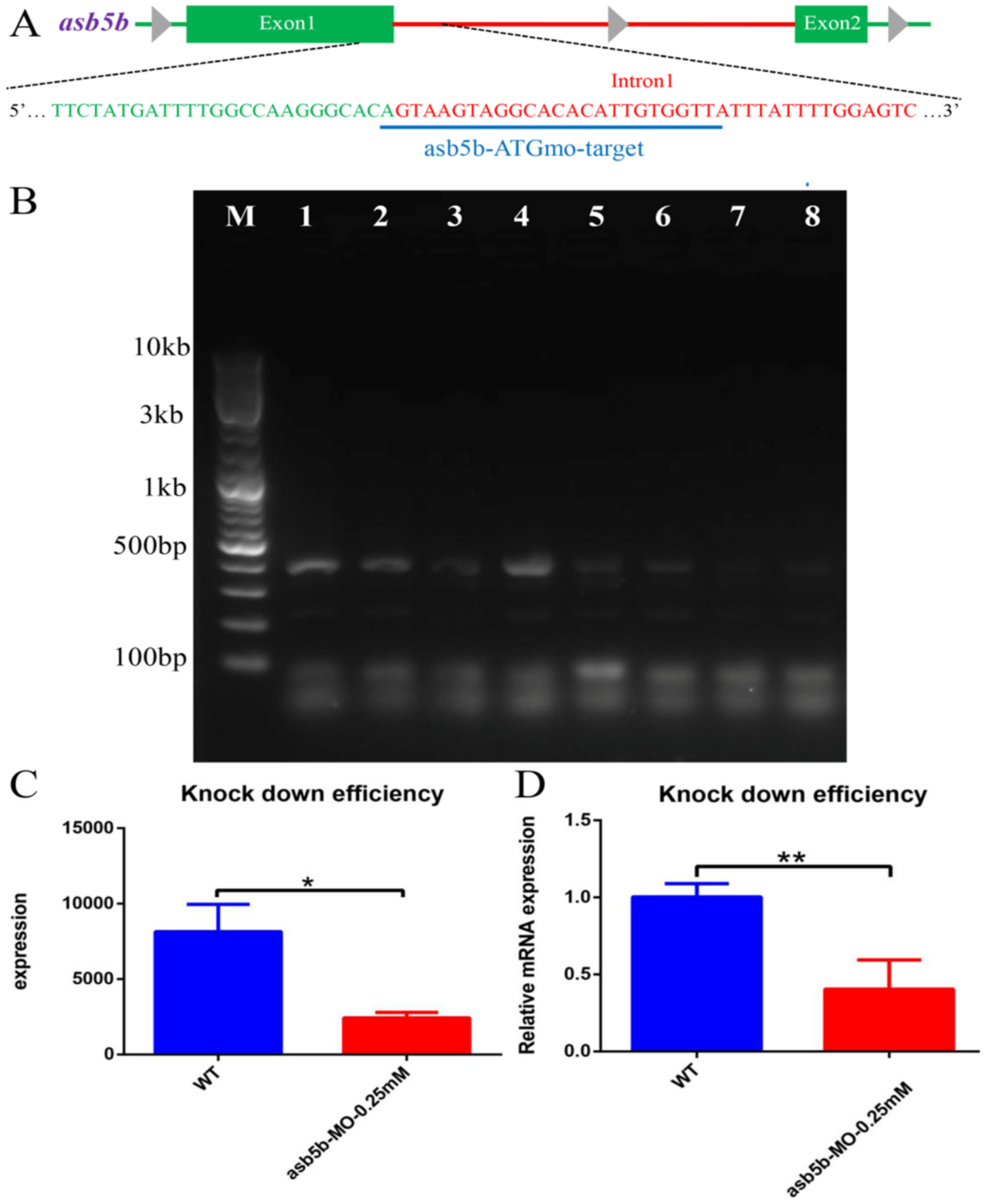

(International Journal of Molecular Sciences, 2023, 16364)

Figure S5. Identification of *asb5b* gene morpholino knockdown in zebrafish. (A) Target site design schematic of *asb5b*-ATGmo. Green represents the exons region, red represents the introns region, and blue represents the target site region. (B) The expression of *asb5b*-mRNA in 48hpf embryos was identified by RT-PCR. M: DNA marker bands; 1-4: Four biological replicates of WT, 5-8: Four biological replicates of *asb5b*-ATGmo-0.25 mM were injected at the WT embryo one-cell stage. (C) Results of gray scale analysis electrophoresis. The results showed that at a low concentration of 0.25 mM, effectiveness knockdown of *asb5b*-mRNA expression levels compared with the WT group. (D) RT-qPCR identified *asb5b*-mRNA expression levels in 48hpf WT embryos after injection of 0.25 mm low concentration *asb5b*-ATGmo. Plotted data represent means ± standard error of the mean (SEM; n = 10, N=4). * $p < 0.05$; ** $p < 0.01$.

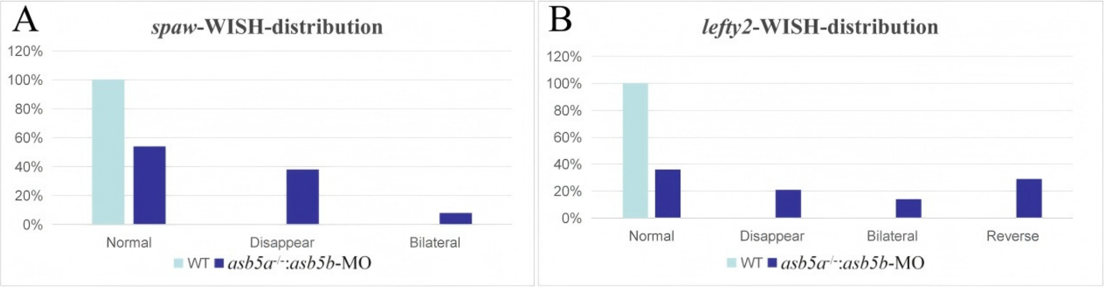

Figure S6. Statistical graphs of WISH results related to genes *spaw* and *lefty*2.
